# SEPTIN9 R106W in a Chinese family with hereditary neuralgic amyotrophy: phenotypic heterogeneity and rehabilitation in a pediatric case

**DOI:** 10.3389/fgene.2026.1909402

**Published:** 2026-08-12

**Authors:** Jing Chen, Shuang Chen, Xin-yi Zhu, Wei Su, Wen-Han Zhang, Dan Gao, Zhi-sheng Liu, Jing-jing Li, Hong-min Zhu

**Affiliations:** 1 Wuhan Children’s Hospital Pediatric Rehabilitation Medicine, Tongji Medical College, Huazhong University of Science and Technology, Wuhan, China; 2 Pediatric Gastroenterology, Wuhan Children’s Hospital, Tongji Medical College, Huazhong University of Science and Technology, Wuhan, China; 3 Department of Radiology, Wuhan Children’s Hospital, Tongji Medical College, Huazhong University of Science & Technology, Wuhan, China; 4 Department of Neurology, Wuhan Children’s Hospital, Tongji Medical College, Huazhong University of Science and Technology, Wuhan, China

**Keywords:** hereditary neuralgic amyotrophy, pain episodes, rehabilitation, Septin9, variable expressivity

## Abstract

**Introduction:**

Hereditary neuralgic amyotrophy (HNA) is a rare autosomal dominant recurrent focal neuropathy characterized by acute episodes of severe neuropathic pain followed by muscle weakness and atrophy, most commonly affecting the brachial plexus. Pathogenic variants in *SEPTIN9* with c.316C>T (p. Arg106Trp; NM_001113491.2) missense mutation corresponding to c.262C>T (p. Arg88Trp; NM_006640.4) constituting a recurrent hotspot that accounts for approximately 55% of HNA families in which a pathogenic *SEPTIN9* variant can be identified. Although well documented in Caucasian and some Asian populations, reports in the Chinese population remain scarce, and the full phenotypic spectrum and optimal management strategy are incompletely defined.

**Methods:**

Pathogenic variants were identified by whole-exome sequencing (WES) of the proband and confirmed by Sanger sequencing in available relatives.

**Results:**

We report a three-generation Chinese pedigree harboring the *SEPTIN9* R106W mutation. The proband, a 34-year-old female, experienced a painless, self-limiting left upper limb weakness at age nine that resolved spontaneously after 6 months, following a 20-year asymptomatic period. She relapsed postpartum at age 29 with bilateral upper limb pain, weakness, and atrophy, resulting in residual neurological deficits. Her 5-year-old daughter presented with infection-triggered classic childhood HNA, exhibiting distinctive facial features (hypertelorism, epicanthal folds, short palpebral fissures, microstomia, and neck webbing), scapular winging, and severe right upper-limb motor impairment. The child showed functional improvement temporally associated with treatment following corticosteroid pulse therapy, neurotrophic support, and a structured, phased rehabilitation protocol. The proband’s father had atypical hand numbness and tremor in young adulthood and later died of systemic amyloidosis at age 66, but his *SEPTIN9* genotype could not be determined because genetic testing was not performed. This pedigree demonstrates marked intrafamilial variable expressivity.

**Discussion:**

This report delineates the broad clinical spectrum associated with the *SEPTIN9* R106W mutation in a Chinese pedigree, spanning from childhood to adulthood. Infection and childbirth were identified precipitating factors. The pronounced phenotypic variability underscores the necessity of early molecular diagnosis and cascade screening. Furthermore, prompt multidisciplinary management combining immunomodulation and structured rehabilitation achieved substantial functional recovery in the pediatric patient, highlighting the critical role of active inter vention in childhood-onset HNA.

## 1 Introduction

Hereditary neuralgic amyotrophy (HNA, OMIM 162100) is a rare autosomal dominant recurrent focal neuropathy clinically characterized by acute, episodic brachial plexus neuropathy (Neubauer et al., 2019). Affected individuals typically experience severe pain in the shoulder and arm, followed by patchy muscle weakness and atrophy. Potential triggers include preceding infections, immune activation, surgeries, pregnancies, deliveries, strenuous exercise, cold exposure, and emotional stress (van Alfen and van Engelen, 2006). This recurrent nature often leads to episodic disability and may result in long-term functional deficits. Globally, approximately 300 HNA families have been reported, with ages of onset ranging from infancy to adulthood (Bhatti et al., 2024). *SEPTIN9* (also historically denoted SEPT9) has been established as the major causative gene for HNA. Among the identified pathogenic variants, the c.316C>T (p. Arg106Trp; NM_001113491.2) missense mutation—corresponding to c.262C>T (p. Arg88Trp; NM_006640.4) in an alternative transcript—represents a recurrent hotspot reported across diverse ethnic populations (Bhatti et al., 2024; Hannibal et al., 2009; Kuhlenbäumer et al., 2005; Ueda et al., 2010). This mutation accounts for approximately 55% of HNA families with identifiable *SEPTIN9* pathogenic variants (Hannibal et al., 2009; Kuhlenbäumer et al., 2005; Meiling et al., 2024). Carriers exhibit striking clinical heterogeneity with variability in triggers, age of onset, symptom severity, and long-term outcomes, likely influenced by modifier genes, environmental factors, and epigenetic mechanisms (Bhatti et al., 2024).

*SEPTIN9* encodes a conserved filament-forming GTPase highly expressed in Schwann cells of the peripheral nervous system. Septin-9 participates in multiple cellular processes, including cytokinesis, vesicular transport, exocytosis, cell polarity, and motility. It interacts with the cytoskeleton—specifically microtubules and actin—thereby promoting asymmetric neurite growth (Füchtbauer et al., 2011). The R106W mutation disrupts microtubule bundling and impairs neurite outgrowth in cellular models (Alkhanjari et al., 2025). Molecular dynamics simulations further suggest that this mutation alters the spatial conformation of the Septin-9 N-terminal proline-rich domain, impairing its binding to microtubules and its bundling capacity (Bhatti et al., 2024). While the genotype–phenotype correlation of the *SEPTIN9* R106W mutation has been relatively well described in Caucasian and some Asian populations, reports within the Chinese population remain exceedingly scarce. Consequently, its penetrance, full clinical spectrum, and potential unique phenotypic subtypes in this population are not yet clearly defined.

In the present study, we report a three-generation Chinese family in which two members carry the same *SEPTIN9* R106W hotspot mutation. This pedigree exhibits remarkable variable expressivity. Notably, the proband’s daughter represents a very young pediatric case of infection-triggered HNA. Additionally, we provide a detailed description of the rehabilitation strategy and functional outcomes in this pediatric patient, offering a potential reference for the management of childhood HNA.

## 2 Materials and methods

### 2.1 Ethical statement

The studies involving humans were approved by the Ethics Committee of Wuhan Children’s Hospital (Grant No. 2022R043-E01). The studies were conducted in accordance with the Declaration of Helsinki and local institutional requirements. Written informed consent was obtained from all adult participants and from the legal guardians of the minor participant for genetic testing, clinical data publication, and publication of identifiable clinical photographs.

### 2.2 Study design and participants

This study was conducted as a case series with familial co-segregation analysis. The pedigree was constructed through detailed family history interviews and clinical examinations. The report focuses on the proband’s daughter, as the proband was in an asymptomatic interval at the time of evaluation and her father was deceased.

### 2.3 Clinical assessment

Neurological examination included comprehensive motor and sensory evaluations, cranial nerve assessment, and musculoskeletal inspection. Electrophysiological studies comprised electromyography (EMG) and nerve conduction studies (NCS) using standard protocols. Imaging studies included magnetic resonance imaging (MRI) of the brachial plexus and shoulder joint. Laboratory investigations included complete blood count, liver and renal function tests, comprehensive immunology panel, and autoantibody screening. Functional assessments were performed using the Gesell Developmental Schedules and the Revised Upper Limb Module (RULM) test.

### 2.4 Genetic analysis

Whole-exome sequencing (WES) was performed on the proband (II-1) to identify pathogenic variants. Sanger sequencing was then used for familial segregation analysis in the proband’s mother (I-2), sister (II-3), and daughter (III-1). The proband’s niece (III-2) was not available for genetic testing.

Genomic DNA was extracted from peripheral blood using the QIAamp DNA Blood Mini Kit (Qiagen). WES was performed using the Agilent SureSelect Human All Exon V6 capture kit and sequenced on the Illumina NovaSeq 6000 platform (150 bp paired-end reads). The sequencing generated 87,061,100 total reads, with a mean target coverage of 134.92×. Coverage at ≥20× was achieved for 98.4% of target regions. Reads were aligned to the GRCh37/hg19 reference genome using BWA-MEM, and variant was called using GATK HaplotypeCaller (v4.1). Variants were annotated with ANNOVAR and filtered against population databases (gnomAD v2.1.1, ExAC) and disease databases (ClinVar, OMIM). Copy number variation analysis was not performed. Sanger sequencing was performed using BigDye Terminator v3.1 on an ABI 3730xl DNA Analyzer.

### 2.5 Rehabilitation protocol

A structured rehabilitation protocol was initiated in the second month of illness, following acute-phase anti-inflammatory therapy. The program adopted a phased, goal-directed strategy:

Phase I (weeks 1–4): Aimed to achieve grade 3 shoulder muscle strength through assisted active range-of-motion exercises, multi-angle isometric contractions, and transcutaneous electrical nerve stimulation (TENS). Concurrently, mirror therapy with biofeedback was employed to reinforce motor intention and promote cortical reorganization.

Phase II (weeks 5–8): Transitioned to strength reinforcement via active isotonic movements, incorporating progressive resistance and endurance training with age-appropriate equipment (e.g., toys, dowels, and graded sandbags).

Phase III (weeks 9–12): Introduced targeted scapular stabilization exercises to enhance glenohumeral stability and correct aberrant compensatory movement patterns.

## 3 Results

### 3.1 Clinical presentations

#### 3.1.1 Proband (II-1, 34-year-old female)

The proband’s first episode occurred at age of 9 years (1999) with sudden, painless weakness of the left upper limb without identifiable precipitating factors. Electromyography (EMG) at that time suggested neurogenic changes. She received intermittent acupuncture and physiotherapy with minimal improvement. Nevertheless, her symptoms resolved entirely after 6 months without residual deficits, followed by a 20-year asymptomatic period.

Her second episode occurred at age 29 years, on the third postpartum day following a vaginal delivery that was converted to caesarean section. In contrast to the initial episode, she experienced severe pain in the right upper limb that subsequently radiated bilaterally. This pain was accompanied by weakness in her right hand, predominantly the thumb, index, and middle finger, and numbness. Magnetic resonance imaging (MRI) of the right upper arm revealed signs of denervation-related muscular changes. EMG confirmed neurogenic damage in the right median nerve and bilateral ulnar nerves. Following supportive treatments including acupuncture, physiotherapy, and oral mecobalamin, her condition partially improved. However, residual deficits persisted, including continuous numbness in the right thumb and mild weakness in the right upper limb.

#### 3.1.2 Proband’s daughter (III-1, 5-year-old female)

The proband’s daughter, a 5-year-old girl, presented with sudden onset intermittent pain in the proximal right upper limb on the third day of a pneumonia infection. The pain was accompanied by progressively worsening right arm weakness, which subsequently led to atrophy of proximal muscles, including the deltoid. No sensory abnormalities were reported.

**FIGURE 1:**
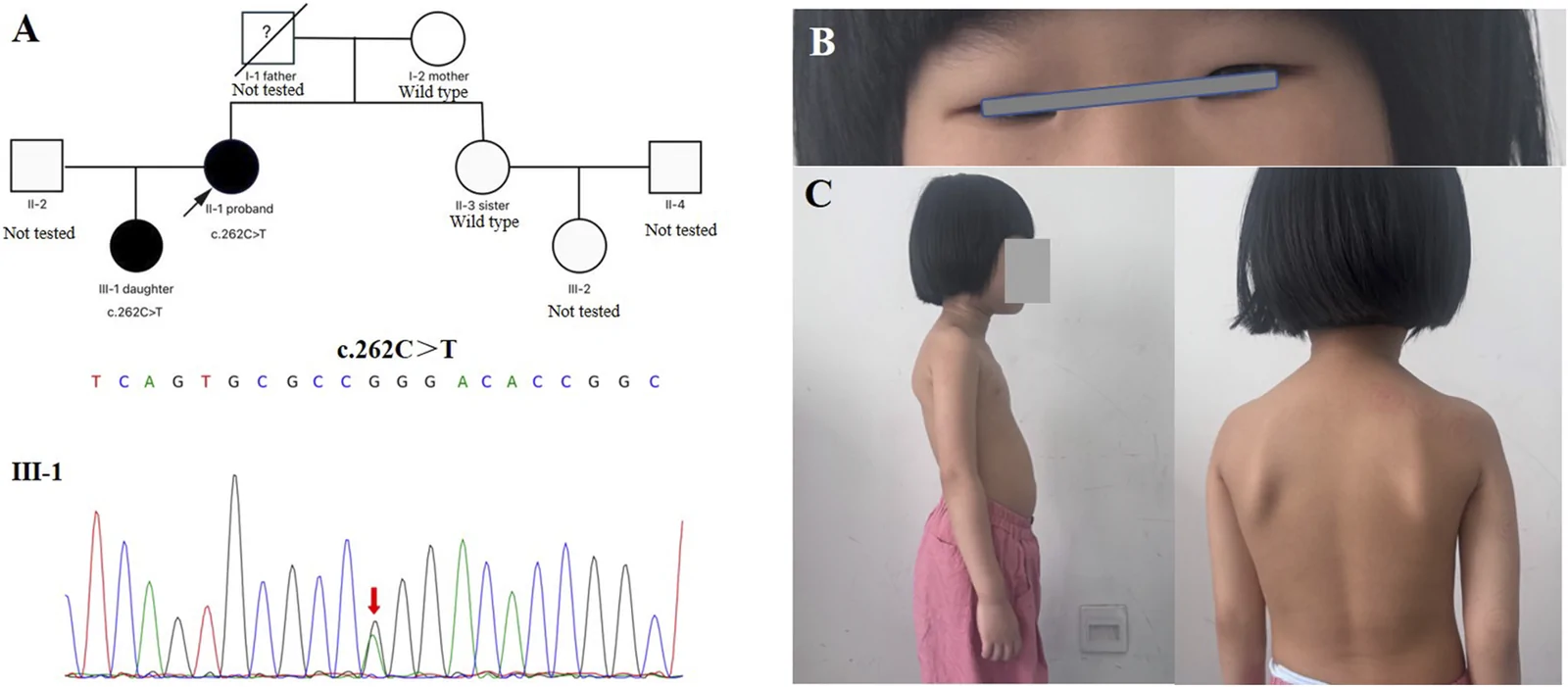
Clinical presentation, pedigree, and genetic confirmation of the Chinese family with SEPTIN9-related hereditary neuralgic amyotrophy **(A)** Pedigree and genetic analysis of the three-generation Chinese family with the SEPTIN9 c.262C>T variant. Squares and circles denote males and females, respectively. Filled symbols indicate affected individuals; open symbols indicate unaffected individuals. The arrow identifies the proband (II-1). The proband (II-1) and her daughter (III-1) both carry the heterozygous c.262C>T variant. The proband’s mother (I-2) and sister (II-3) are wild type. The proband’s niece (III-2) was not tested. The proband’s father (I-1) is indicated with a question mark as his genotype is unknown. **(B)** Clinical photographs of the proband’s daughter (III-1) at age 5 years showing distinctive facial features, including hypertelorism, epicanthal folds, short palpebral fissures, microstomia, and neck webbing. **(C)** Posterior and lateral views demonstrating asymmetric shoulder posture with right shoulder drooping (left panel), prominent right scapular winging (middle panel), and right deltoid muscle atrophy (right panel).

Physical examination revealed an alert child with distinctive facial features, including hypertelorism, epicanthal folds, short palpebral fissures, microstomia, and neck webbing (Figure 1B). Intelligence and language development were age appropriate. Musculoskeletal findings included prominent scapular winging and anterior pelvic tilt (Figure 1C). Anthropometric measurements showed bilateral upper arm circumference of approximately 16.5 cm, with evident atrophy of the right deltoid muscle and restricted active movement of the right upper limb (Figure 1C). Muscle strength and tone in the left upper limb were normal. Lower limb strength was graded 5/5 bilaterally with normal tone, preserved patellar tendon reflexes, and absence of pathological signs.

**FIGURE 2:**
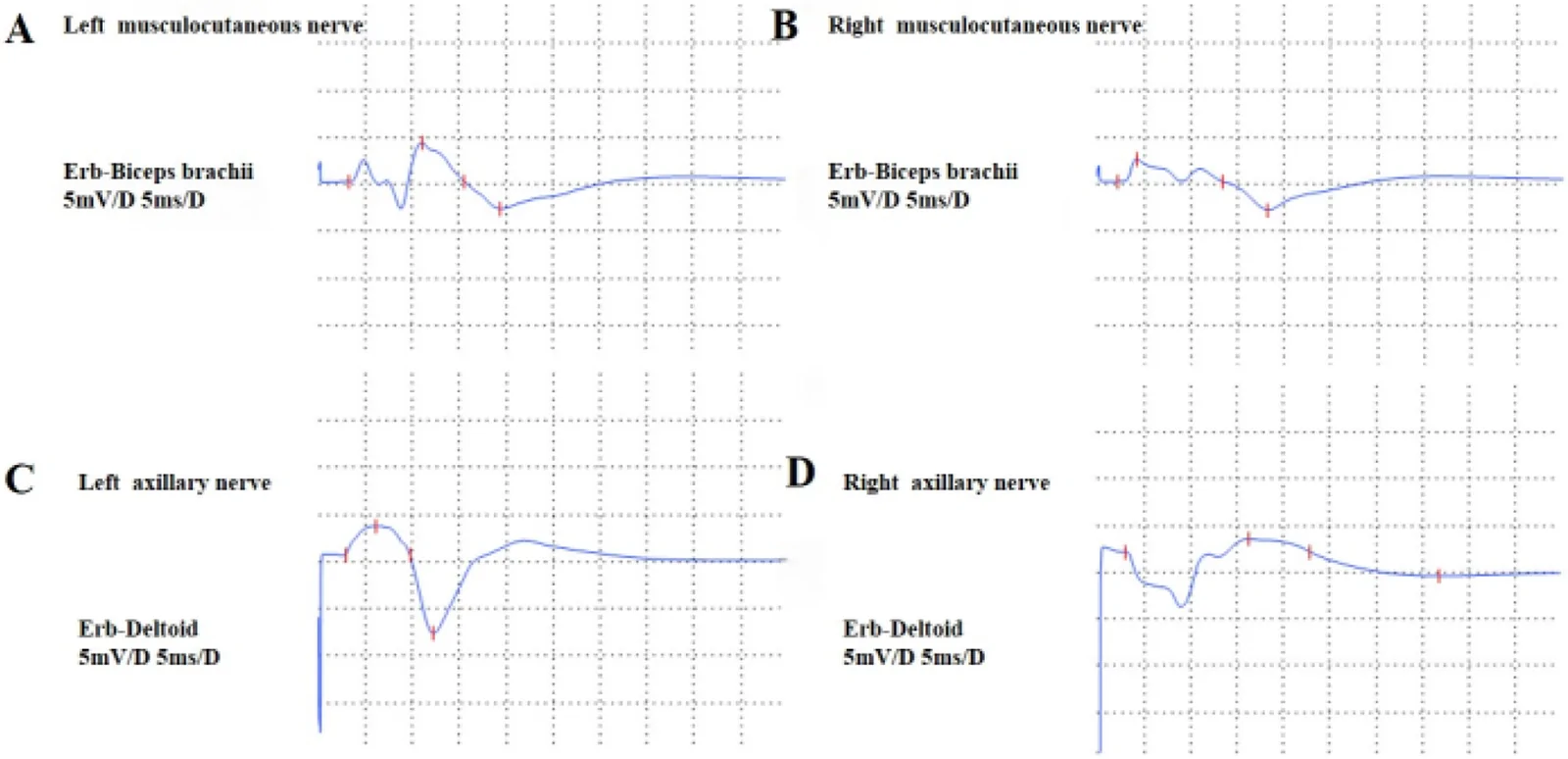
Bilateral musculocutaneous and axillary nerve motor conduction studies. **(A)** Left musculocutaneous nerve motor conduction study showing normal compound muscle action potential (CMAP) amplitude and latency. Stimulation was performed at Erb’s point, with recording from the biceps brachii muscle. Calibration: 5 mV/division, 5 m/division. **(B)** Right musculocutaneous nerve motor conduction study demonstrating markedly reduced CMAP amplitude compared with the contralateral side, consistent with axonal loss. Stimulation was performed at Erb’s point, with recording from the biceps brachii muscle. Calibration: 5 mV/division, 5 m/division. **(C)** Left axillary nerve motor conduction study showing normal CMAP amplitude and latency. Stimulation was performed at Erb’s point, with recording from the deltoid muscle. Calibration: 5 mV/division, 5 m/division. **(D)** Right axillary nerve motor conduction study demonstrating reduced CMAP amplitude compared with the contralateral side, consistent with neurogenic damage. Stimulation was performed at Erb’s point, with recording from the deltoid muscle. Calibration: 5 mV/division, 5 m/division.

**FIGURE 3:**
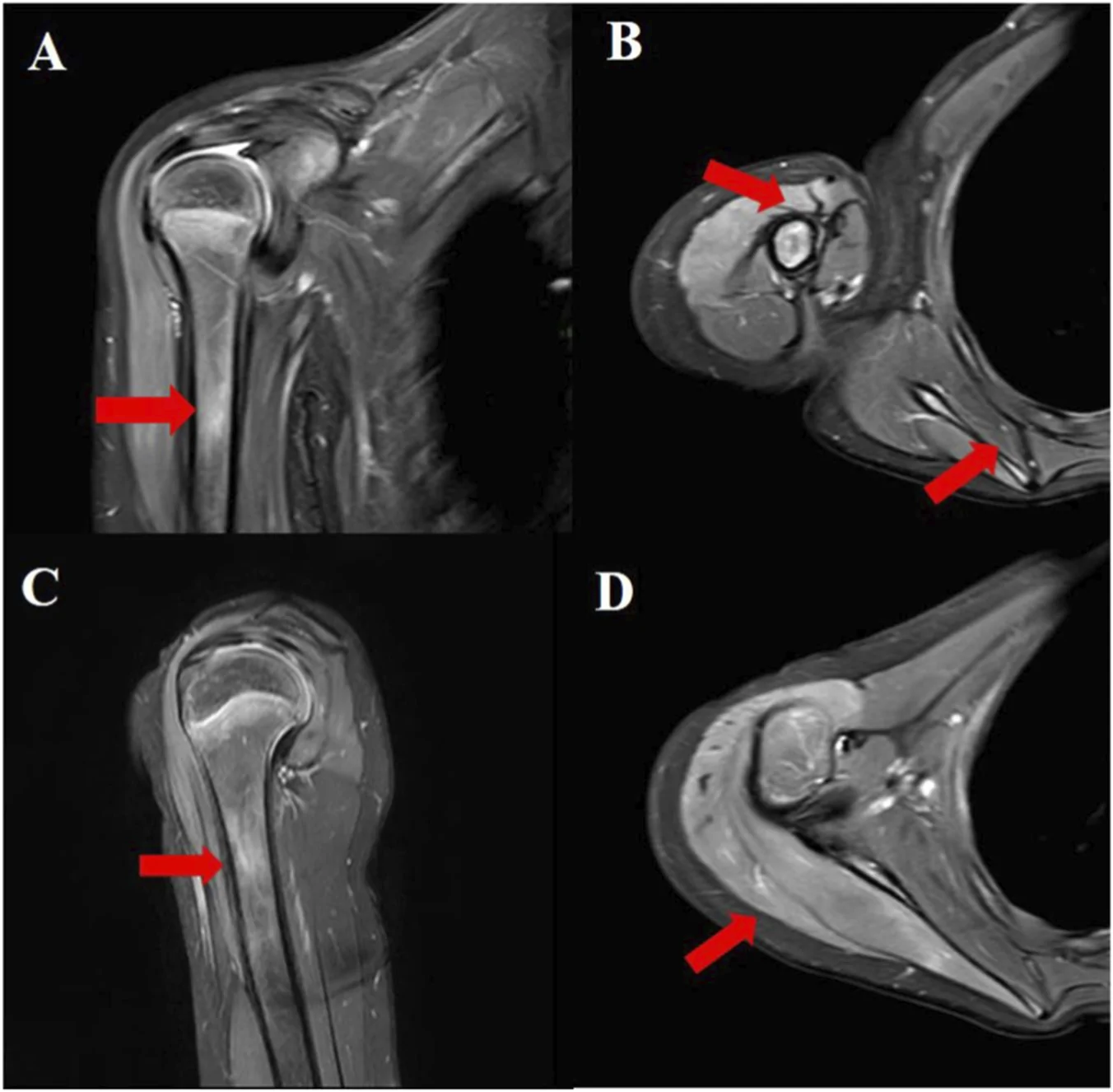
Magnetic resonance imaging (MRI) of the right shoulder and brachial plexus at initial presentation. **(A)** Coronal T2-weighted fat-suppressed image showing hyperintense signal (arrow) within the proximal right humeral diaphysis and metaphysis, consistent with bone marrow edema. **(B)** Axial T2-weighted fat-suppressed image demonstrating mild joint effusion (upper arrow) and edema of the surrounding musculature (lower arrow), including the deltoid and rotator cuff muscles. **(C)** Sagittal T2-weighted fat-suppressed image revealing hyperintense signal (arrow) in the proximal humeral metaphysis, suggestive of bone marrow edema extending along the humeral shaft. **(D)** Axial T2-weighted fat-suppressed image at a different level showing soft tissue edema (arrow) surrounding the right shoulder joint, with involvement of the subcutaneous tissues and adjacent musculature.

Neurophysiological studies revealed reduced motor conduction amplitudes in the right musculocutaneous and axillary nerves compared to the contralateral side, together with fibrillation potentials in the right deltoid muscle, consistent with neurogenic damage (Figure 2). Brachial plexus MRI demonstrated segmental thickening and edema of the right axillary nerve. Shoulder MRI revealed right joint effusion, swelling of the surrounding muscle layers, and bone marrow edema in the proximal right humerus (Figure 3). Complete blood count, liver and renal function tests, comprehensive immunology panel, and autoantibody screening were all unremarkable. Functional assessment using the Gesell Developmental Schedules indicated delayed gross motor development. The Revised Upper Limb Module (RULM) test demonstrated severely impaired function in the right upper limb, in stark contrast to normal function on the left.

#### 3.1.3 Proband’s father (I-1, deceased)

The proband’s father never exhibited classic HNA symptoms. Around the age of 30, he developed hand numbness and tremors, which resolved spontaneously without medical intervention. At age 60, he was diagnosed with systemic amyloidosis with multi-organ dysfunction, including proteinuria, atypical myeloma, hepatomegaly, purpura of the head and neck, arm numbness, carpal tunnel syndrome, and cardiopulmonary dysfunction. He died at age 66 years from multi-organ failure. Genetic testing was not performed.

### 3.2 Genetic test results

Singleton whole-exome sequencing (WES) was performed on the proband to identify pathogenic variants. Sanger sequencing was used for familial co-segregation analysis in the proband’s mother, sister, and daughter. The results demonstrated that the proband (II-1) and her daughter (III-1) carried a heterozygous mutation in *SEPTIN9*: c.262C>T (p. Arg88Trp; NM_006640.4), corresponding to the well-documented R106W mutation (c.316C>T, p. Arg106Trp; NM_001113491.2) (Figure 1A). The proband’s mother (I-2) and sister (II-3) were wild type at this locus. The proband’s niece (III-2) was not tested for this variant. According to the ACMG/AMP guidelines, this variant was classified as pathogenic (PP1_Strong: co-segregation with disease in this family; PM2: absent from population databases; PS3_Moderate: functional studies demonstrating altered Rho/Rhotekin signaling; PS4_Moderate: well-established pathogenicity in multiple independent families; PP3: multiple computational predictions of deleterious effect).

### 3.3 Treatment and rehabilitation outcomes

The pediatric patient received acute-phase anti-inflammatory therapy with intravenous dexamethasone (9 mg per dose once daily for 5 days, followed by 4 mg per dose once daily for 2 days; total 7 days), then transitioned to oral dexamethasone acetate tablets (0.75 mg per tablet: 3 tablets twice daily for 2 days, then 2 tablets once daily for 1 day, then 1 tablet once daily for 1 day; total 6 days). She also received mecobalamin, initially as intravenous injection (0.5 mg per dose once daily for 9 days), followed by oral mecobalamin tablets (0.5 mg per dose twice daily for 94 days), and intramuscular mouse nerve growth factor (30 μg per dose once daily for 74 days). All medications and structured rehabilitation protocol were initiated approximately 6 weeks after symptom onset. Baseline functional assessments (performed at treatment initiation): right RULM score 5; Gesell right fine motor developmental quotient (DQ) 32, and ADL score 75. Following 12 weeks of integrated rehabilitation, repeat assessments showed: RULM score 34; Gesell right fine motor DQ 85. The patient demonstrated improvement in right upper-limb strength from Medical Research Council (MRC) grade 2 to grade 4. At the 1-year follow-up, she remained free from pain and disease recurrence. Complete rehabilitation outcome data are presented in Table 1.

**TABLE 1:** Shoulder function and ADL before and after combined therapy in a child with HNA.

Assessment item		Pre-treatment	Post-treatment (1 month)	Post-treatment (3 months)	1-year follow-up
Key Muscle Strength (Right) (MRC grade)	Deltoid	2	3	4	4
	Supraspinatus	2	3	4	4
	Pectoralis major (clavicular part)	2	3	4	4
	Latissimus dorsi	2	3	4	4
	Infraspinatus and Teres minor	2	3	4	4
Range of Motion (ROM) (Right)	Shoulder Flexion	30°	150°	180°	180°
	Shoulder Extension	10°	30°	45°	45°
	Shoulder Abduction	15°	145°	180°	180°
	Shoulder Adduction	25°	30°	45°	45°
	Shoulder Horizontal Adduction	Unable to complete	110°	135°	135°
	Shoulder Horizontal Abduction	Unable to complete	70°	90°	90°
	Shoulder Internal Rotation	80°	80°	80°	80°
	Shoulder External Rotation	60°	80°	80°	80°
Shoulder Muscle Endurance (Right)	Shoulder Abduction Holding Time	0s	60s	120s	120s
	Shoulder Flexion Holding Time	0s	60s	120s	120s
Pain Assessment	FPS-R	4	2	0	0
RULM	Left	34	34	34	34
	Right	5	24	34	34
Gesell Fine Motor Score	Right	32	-	85	83
ADL Score	​	75	85	100	100

Abbreviations: ADL, activities of daily living; FPS-R, Faces Pain Scale-Revised; MRC, medical research council; RULM, revised upper limb module.

## 4 Discussion

HNA is a disabling autosomal dominant peripheral neuropathy. Its classic neurological presentation encompasses sudden, severe shoulder and arm pain followed by muscle weakness, atrophy, and possible sensory disturbances or frozen shoulder. Affected individuals may also exhibit distinctive facial features such as hypotelorism, epicanthal folds, and thickened neck creases (Musa et al., 2021; Abdouni and Viamont-Guerra, 2024; Bosisio et al., 2025). Onset typically occurs in childhood, with earlier onset correlating with a higher risk of recurrence (Bosisio et al., 2025). Common triggers include infection, strenuous exercise, surgery, and pregnancy, reported in approximately 53.2% of acute episodes (van Alfen, 2007; van Alfen et al., 2009; Serin et al., 2019). Recurrence affects about 75% of patients, with an average interval of 6 years. Interictal periods vary significantly, ranging from complete symptom resolution to chronic neurological deficits (Abdouni and Viamont-Guerra, 2024; Kim et al., 2021). Electrophysiological studies consistently reveal neurogenic damage predominantly in the upper limbs, with the lower limbs rarely involved (Bosisio et al., 2025; Kim et al., 2021). In 2005, mutations in *SEPTIN9* were identified as the principal cause of HNA. The c.316C>T (p. Arg106Trp; NM_001113491.2) missense mutation—corresponding to c.262C>T (p. Arg88Trp; NM_006640.4)—represents a recurrent hotspot reported across diverse ethnic populations and accounts for approximately 55% of HNA families in which a pathogenic SEPTIN9 variant can be identified (Hannibal et al., 2009; Kuhlenbäumer et al., 2005; Meiling et al., 2024). Table 2 summarizes the clinical features and treatment outcomes of previously reported carriers of this variant.

**TABLE 2:** Clinical characteristics of previously reported carriers of the SEPTIN9 c.262C>T (p. Arg88Trp; NM_006640.4)/c.316C>T (p.Arg106Trp; NM_001113491.2) variant and the present family.

References	Case	Sex	Age at onset (yr)	Episodes (n)	Affected site	Clinical manifestations	Dysmorphic features	Treatment	Prognosis
Present family	II-1 (proband)	F	9	2	Left upper	1st: painless weakness, resolved; 2nd: pain, weakness, atrophy, residual numbness	Short palpebral fissures	Acupuncture, physiotherapy, mecobalamin	Complete recovery (1st), partial (2nd)
	III-1 (daughter)	F	5	1	Right upper	Pain, weakness, deltoid atrophy, scapular winging	Hypertelorism, epicanthal folds, short palpebral fissures, microstomia, neck webbing	Dexamethasone, mecobalamin, mNGF, phased rehabilitation	Substantial functional recovery
China (Zhao et al., 2020)	Proband	M	4	1	Bilateral upper limbs	Pain, weakness (wrist drop, distal > proximal), muscle atrophy; no sensory loss; no trigger; non-recurrent	Forearm annular skin folds	N/A	N/A
Japan (Ueda et al., 2010)	I-1	F	28	2	Upper limbs (left > right)	Pain (94% of attacks), weakness, moderate sensory loss (left upper); triggers: exercise, delivery, trauma, injections; recurrent	Hypotelorism, short stature, skin wrinkles, ear deformities (6/6 patients)	N/A	Complete recovery in 41% of attacks
	II-5	M	12	3	Upper and lower limbs (left >> right)	Pain, weakness, mild sensory loss; recurrent		N/A	N/A
	II-6	F	22	2	Upper limbs	Pain, no weakness/atrophy/sensory loss; recurrent		N/A	N/A
	III-1	M	18	4	Upper limbs (left > right)	Pain, weakness, no sensory loss; recurrent		N/A	N/A
	III-2	M	11	4	Left upper limb	Pain, weakness, moderate sensory loss; recurrent		N/A	N/A
	III-12	F	19	2	Left upper limb	Pain, weakness, mild sensory loss; recurrent		N/A	N/A
Lebanon (Neubauer et al., 2019)	Daughter (proband)	F	2	3	1st: left upper; 2nd-3rd: right upper	Pain (all attacks), weakness (wrist drop, arm weakness), muscle atrophy (left hand, right deltoid), sensory loss (2nd attack); trigger: vigorous exercise; recurrent	Epicanthus, macroglossia, 4 fingerprint ridges	Acute NSAIDs + physiotherapy	Partial recovery (residual weakness after 2nd attack)
	Father	M	14	2	Bilateral upper limbs	Pain (2 weeks), weakness, muscle atrophy (shoulders), sensory loss (proximal arm/elbow); trigger: vigorous exercise; recurrent	Epicanthus	N/A	Partial recovery (sensory loss, shoulder atrophy)
	Brother	M	6	1	Right upper limb	Pain (2–3 weeks), weakness, muscle atrophy (right shoulder/arm), no sensory loss; trigger: vigorous exercise; non-recurrent	N/A	N/A	Complete recovery
Japan (Ueno et al., 2023)	Daughter (proband)	F	17	7	1st-6th: right upper; 7th: left upper	Pain (all), weakness (1st-6th right, 7th left), sensory loss (7th: mild left thumb/forearm); triggers: unknown (1st-6th), golf injury (7th); recurrent	Hypotelorism, high-arched palate	1st-6th: none; 7th: analgesics, carpal tunnel release (ineffective), methylprednisolone pulse	Mostly recovered, residual left thumb sensory deficit
	Brother	M	Childhood	2	Left upper (axillary, posterior interosseous)	N/A	N/A	Surgery (unspecified)	N/A
India (Bhatti et al., 2024)	I-2	M	20	1	Right upper limb	Mild pain, proximal weakness, no atrophy/sensory loss; trigger: minor blunt trauma; non-recurrent	Hypotelorism, epicanthus, neck skin folds	N/A	Complete recovery
	II-3	M	17	1	Bilateral upper (asymmetric)	Mild pain, asymmetric weakness, no atrophy/sensory loss; trigger: upper respiratory infection; non-recurrent	Hypotelorism, epicanthus, neck skin folds	N/A	Complete recovery
	I-4	M	20	1	Left upper limb	Mild pain, proximal weakness, no atrophy/sensory loss; no trigger; non-recurrent	Hypotelorism, epicanthus, neck skin folds	N/A	Complete recovery
	III-4	M	15	1	Right upper limb	Mild pain, proximal weakness, no atrophy/sensory loss; trigger: upper respiratory infection; non-recurrent	Hypotelorism, epicanthus, neck skin folds	N/A	Complete recovery
	III-10	F	13	1	Right upper limb	Mild pain, distal weakness, muscle atrophy (right upper), no sensory loss; trigger: examination stress; non-recurrent	Hypotelorism, epicanthus, neck skin folds	N/A	Complete recovery
	III-9 (proband)	F	12	3	1st: right upper; 2nd: asymmetric bilateral; 3rd: right upper	Pain (mild to severe), asymmetric weakness, muscle atrophy (2nd: left shoulder/arm and right distal; 3rd: left proximal), sensory loss (2nd: local); triggers: blunt trauma, stress, unknown; recurrent	Hypotelorism, epicanthus, neck skin folds	1st: none; 2nd: methylprednisolone pulse; 3rd: long-term steroids	Partial recovery
Australia (Chuk et al., 2016)	Proband	M	5	2	1st: right upper; 2nd: left upper	Severe pain, weakness (right shoulder abduction limited), muscle atrophy (1st: supraspinatus/infraspinatus), numbness (1st); trigger: gastroenteritis; recurrent	Mandibular hypoplasia, ectropion, midface hypoplasia, microtia, pectus carinatum, forearm skin folds	1st: IVIg 2 g/kg	Improvement after IVIg, residual mild weakness
Italy (Bosisio et al., 2025)	Daughter (proband)	F	5	2	Right upper limb	Pain (weeks), weakness (right arm elevation difficulty), muscle atrophy (bilateral shoulder, pectoralis, right > left), no sensory loss; no trigger; recurrent	Hypotelorism, small palpebral fissures, epicanthus, long nasal bridge, neck skin folds	N/A	Partial recovery
	Father	M	10	2	1st: right upper; 2nd: left hand	Pain, weakness (1st right upper, 2nd left hand), muscle atrophy (2nd: mild thenar), sensory changes (2nd, unspecified); trigger: sleep compression (2nd); recurrent	N/A	N/A	Partial recovery
Israel (Leshinsky-Silver et al., 2013)	III-1 (proband)	M	Birth	5	Left hand, right shoulder/arm, vocal cords	Severe pain (left arm), weakness (left arm, later right arm); no atrophy/sensory data reported; recurrent	Short stature, hypotelorism, small mouth, forearm circular skin folds	IVIg at 2.5 years for vocal cord palsy	Vocal cord palsies recovered; limb weakness partial
Austria (Lac et al., 2008)	Brother (proband)	M	2.5	N/A	Right arm	Pain (right arm); other manifestations not reported	Cleft palate, narrow palpebral fissures, thin eyebrows, mild ptosis, epicanthus, small mouth, ear malformations, circular skin folds	N/A	N/A
	Sister	F	2.5	N/A	Right elbow	Persistent pain (right elbow), mild weakness (right elbow)	Narrow palpebral fissures, thin eyebrows, mild ptosis, epicanthus, small mouth, ear malformations, circular skin folds	N/A	N/A
	Father	M	10	3	Left shoulder/arm, diaphragm, vocal cord	Pain, weakness (left shoulder/arm), muscle atrophy (arm after last attack); recurrent	Narrow face, hypotelorism, bifid uvula	N/A	Partial recovery
	Grandmother	F	N/A	≥3	Left shoulder/arm	Pain, weakness; recurrent	N/A	N/A	N/A

Abbreviations: Epis., episodes; F, female; IVIg, intravenous immunoglobulin; M, male; mNGF, mouse nerve growth factor; MRC, medical research council; N/A, not available; RULM, revised upper limb module; y, year.

Complete recovery: full muscle strength recovery (MRC, grade 5/5) with no residual sensory deficits or functional impairment. Partial recovery: residual muscle weakness (MRC, grade ≤4) and/or persistent sensory deficits.

### 4.1 Clinical phenotype and genotype-phenotype correlation

The clinical manifestations in our pedigree largely align with this classic HNA phenotype but also reveal distinctive features that expand the clinical spectrum associated with the SEPTIN9 R106W variant. The proband’s initial episode was painless and self-limiting, followed by an unusually long 20-year asymptomatic period, whereas her second episode, triggered by childbirth, was severe and resulted in residual deficits. Her 5-year-old daughter represents a notably young pediatric case of infection-triggered HNA. The proband’s father presented with atypical hand symptoms and later died of systemic amyloidosis. In the absence of genetic testing, no conclusions regarding a relationship to SEPTIN9 can be drawn. Systemic amyloidosis is a known cause of carpal tunnel syndrome and peripheral neuropathy (Donnelly et al., 2025), which may explain his clinical presentation independently of HNA. Episodes with clear triggers were more likely to cause severe symptoms and residual dysfunction, suggesting that the SEPTIN9 mutation-related phenotype is modulated by precipitating factors, age, and individual constitutional factors. The marked intrafamilial variability expressivity, ranging from a painless, self-limited episode to a painful, debilitating adult-onset recurrence, underscores the complex expression of the SEPTIN9 R106W variant. This complexity likely involves an interplay between the genetic mutation, age-dependent physiological changes, and environmental triggers. The R106W mutation disrupts the cytoskeletal regulatory function of the Septin-9 protein, increasing neural susceptibility (Kuzmić et al., 2022). However, the wide clinical variability supports a multifactorial threshold model, in which an additional insult, such as an immune-mediated inflammatory response triggered by infection or childbirth, may precipitate acute neuritis in genetically predisposed individuals (van Alfen, 2011). This pedigree exhibits marked intergenerational variable expressivity, but does not demonstrate incomplete penetrance, as both molecularly confirmed carriers (the proband and her daughter) are clinically affected, and no confirmed asymptomatic carrier was identified. Similarly, the limited number of meioses (only one confirmed parent-child transmission) precludes any meaningful assessment of genetic anticipation.

A recent multicenter study of 12 patients with SEPTIN9-related HNA reported substantial diagnostic delays (median 18 years), underscoring that symptoms often manifest before genetic testing is pursued and that the condition remains underrecognized (Theuriet et al., 2026). Although not highly sensitive or specific, certain clinical features may prompt suspicion of a SEPTIN9-related etiology in patients with neuralgic amyotrophy. These features include young age, dysmorphic features (particularly hypertelorism, epicanthal folds, and facial dysmorphism), recurrent episodes, sensory symptoms, and distal nerve involvement in the upper limbs.

### 4.2 Molecular interpretation and established pathogenicity

At the molecular level, *SEPTIN9* encodes a filament-forming GTPase that undergoes extensive alternative splicing, generating multiple tissue-specific transcript variants (e.g., *SEPTIN9*_v1 through v6), all of which share a conserved C-terminal GTP-binding domain. The R106W mutation resides in the N-terminal proline-rich domain, which contains a microtubule-binding repeat motif (K/R-x-x-E/R/K-R-x-E) that interacts with the acidic C-terminal tail of β-tubulin to mediate microtubule bundling. Molecular dynamics simulations and cellular studies indicate that the Arg106Trp substitution induces conformational changes that impair this microtubule-binding and bundling capacity, leading to reduced neurite outgrowth and asymmetric growth (Bai et al., 2013). Additionally, HNA-associated N-terminal mutations disrupt the co-localization of Septin-9 with Septin-4 and Septin-11 and interfere with Rho/Rhotekin-mediated regulation of septin-containing filaments. Because alternative splicing generates isoforms that differ in their N-terminal domains, only a subset of Septin-9 polypeptides is affected by the R106W mutation, which may partly explain the observed tissue-specific and interindividual phenotypic variability. Notably, under hypoxic conditions, the c.262C>T variant has been reported to alter the translation efficiency of the *SEPTIN9*_v4 transcript (McDade et al., 2007). Collectively, these data suggest that the N-terminal domain of Septin-9 is critical for cytoskeletal interactions and septin complex assembly, and that perturbation of these functions by the R106W mutation contributes to HNA pathogenesis, although the precise pathophysiological cascade remains incompletely understood.

The variant identified in this family—c.316C>T (p.Arg106Trp; NM_001113491.2), corresponding to c.262C>T (p.Arg88Trp; NM_006640.4)—is a well-established pathogenic variant. According to ACMG guidelines, it was classified as pathogenic based on evidence criteria PP1_Strong + PM2 + PS3_Moderate + PS4_Moderate + PP3 (as per the proband’s original WES report). Thus, our study does not newly establish pathogenicity of this variant but rather provides detailed clinical characterization and longitudinal management experience in a Chinese pedigree, contributing to the broader understanding of its phenotypic spectrum and natural history.

### 4.3 Treatment and rehabilitation considerations

Given the potential for severe residual deficits in recurrent episodes, early therapeutic intervention is critical. In this pedigree, the pediatric patient received acute-phase anti-inflammatory therapy with intravenous dexamethasone, oral mecobalamin, and intramuscular mouse nerve growth factor. Rehabilitation was initiated 2 weeks after symptom onset following resolution of acute pain, and the patient’s functional improvement was temporally associated with this combined regimen.

Glucocorticoid pulse therapy likely dampens immune-mediated neuroinflammation and mitigates secondary axonal damage, whereas phased rehabilitation promotes functional reorganization through neuroplasticity (Druzhinina et al., 2025). Although HNA is often considered self-limiting, corticosteroid administration during the acute painful phase may shorten the disease course and improve muscle strength recovery (van Alfen et al., 2009; van Alfen, 2011). Conversely, delayed treatment may lead to irreversible motor unit loss. For typical brachial plexitis, non-surgical comprehensive management combining immunomodulation and rehabilitation offers a superior risk–benefit profile compared with surgical decompression, which should be reserved for specific distal entrapment neuropathies (Rotondo et al., 2020). Pain management remains a primary therapeutic goal; corticosteroids are commonly used in the acute phase to abbreviate pain duration, whereas chronic pain and persistent paralysis warrant referral to physical medicine and rehabilitation specialists. For patients with concomitant phrenic nerve palsy, specialized respiratory consultation and non-invasive nocturnal positive pressure ventilation may be beneficial. Although the child in this report experienced no significant pain, these measures retain important reference value for the broader management of HNA. Furthermore, reports of HNA triggered by vaccination suggest that excessive immune activation constitutes a key pathological link, indirectly supporting immunosuppressive therapy in acute attacks (Serin et al., 2019; Druzhinina et al., 2025). Therefore, for acute HNA attacks in children, early recognition and implementation of glucocorticoid-based immunomodulation supplemented with systematic rehabilitation training constitute a key strategy for optimizing long-term neurological prognosis.

It is important to note that because this is an uncontrolled single-patient observation and spontaneous recovery is part of the natural history of neuralgic amyotrophy, a causal relationship between the interventions and the observed functional recovery cannot be established. The improvement should be interpreted as temporally associated with, rather than caused by the treatment and rehabilitation protocol.

### 4.4 Limitations

The main limitations of this study include the following: First, this report is based on a single pedigree with only two molecularly confirmed affected individuals, which limits the generalizability of some unique phenotypes. Second, the original Sanger sequencing trace files for the wild-type family members were unavailable due to laboratory archiving constraints; their genotypes are reported based on documented clinical test records rather than raw chromatograms. Third, the proband’s father was not genetically tested, and his clinical history was obtained retrospectively. Therefore, his carrier status remains unknown, and his systemic amyloidosis cannot be attributed to the SEPTIN9 variant. Fourth, the lack of functional experimental data specifically addressing the molecular basis of the phenotypic heterogeneity observed in this family prevents a comprehensive understanding of how the mutation leads to different clinical presentations across individuals and generations. Fifth, the rehabilitation outcomes were assessed primarily using clinical functional scales; more objective quantitative measures (e.g., dynamometry, motion analysis) would strengthen future evaluations.

In conclusion, this study provides a detailed clinical characterization of a Chinese pedigree with the SEPTIN9 R106W variant and describes a structured rehabilitation protocol in a pediatric case. Our findings underscore the importance of early genetic diagnosis, family cascade screening, and prompt multidisciplinary intervention including immunomodulation and structured rehabilitation to optimize neurological outcomes in pediatric HNA. While the variant is well-established as pathogenic, our report contributes valuable information on the phenotypic spectrum, intrafamilial variability, and practical management of this rare condition in the Chinese population.

## Data Availability

The original contributions presented in the study are included in the article/supplementary material, further inquiries can be directed to the corresponding authors.
